# Housing Temperature Impacts the Systemic and Tissue‐Specific Molecular Responses to Cancer in Mice

**DOI:** 10.1002/jcsm.13781

**Published:** 2025-04-16

**Authors:** Andrea Irazoki, Emma Frank, Tang Cam Phung Pham, Jessica L. Braun, Amy M. Ehrlich, Mark Haid, Fabien Riols, Camilla Hartmann Friis Hansen, Anne‐Sofie Rydal Jørgensen, Nicoline Resen Andersen, Laura Hidalgo‐Corbacho, Roberto Meneses‐Valdes, Mona Sadek Ali, Steffen Henning Raun, Johanne Louise Modvig, Samantha Gallero, Steen Larsen, Zach Gerhart‐Hines, Thomas Elbenhardt Jensen, Maria Rohm, Jonas T. Treebak, Val Andrew Fajardo, Lykke Sylow

**Affiliations:** ^1^ Department of Biomedical Sciences, Faculty of Health and Medical Sciences University of Copenhagen Copenhagen Denmark; ^2^ Department of Kinesiology, Faculty of Applied Health Sciences, Cairns Family Health and Bioscience Research Complex Brock University Niagara Region Ontario Canada; ^3^ Novo Nordisk Foundation Center for Basic Metabolic Research, Faculty of Health and Medical Sciences University of Copenhagen Copenhagen Denmark; ^4^ Metabolism & Proteomics Core, Helmholtz Center Munich German Research Center for Environmental Health Neuherberg Germany; ^5^ Section of Experimental Animal Models, Department of Veterinary and Animal Sciences University of Copenhagen Copenhagen Denmark; ^6^ Department of Nutrition, Exercise and Sport University of Copenhagen Copenhagen Denmark; ^7^ Clinical Research Centre Medical University of Bialystok Bialystok Poland; ^8^ Institute of Sports Medicine Copenhagen, Department of Orthopedic Surgery M Copenhagen University Hospital – Bispebjerg and Frederiksberg Copenhagen Denmark; ^9^ Institute for Diabetes and Cancer Helmholtz Center Munich Neuherberg Germany; ^10^ Joint Heidelberg‐IDC Translational Diabetes Program, Inner Medicine 1 Heidelberg University Hospital Heidelberg Germany; ^11^ German Center for Diabetes Research (DZD) Neuherberg Germany

**Keywords:** bioenergetics, cancer cachexia, cold‐induced stress, thermogenic tissues, thermoneutrality

## Abstract

**Background:**

Cancer cachexia, affecting up to 80% of patients with cancer, is characterized by muscle and fat loss with functional decline. Preclinical research seeks to uncover the molecular mechanisms underlying cachexia to identify potential targets. Housing laboratory mice at ambient temperature induces cold stress, triggering thermogenic activity and metabolic adaptations. Yet, the impact of housing temperature on preclinical cachexia remains unknown.

**Methods:**

Colon 26 carcinoma (C26)‐bearing and PBS‐inoculated (Ctrl) mice were housed at standard (ST; 20°C–22°C) or thermoneutral temperature (TN; 28°C–32°C). They were monitored for body weight, composition, food intake and systemic factors. Upon necropsy, tissues were weighed and used for evaluation of ex vivo force and respiration, or snap frozen for biochemical assays.

**Results:**

C26 mice lost 7.5% body weight (*p* = 0.0001 vs. Ctrls), accounted by decreased fat mass (−35%, *p* < 0.0001 vs. Ctrls), showing mild cachexia irrespective of housing temperature. All C26 mice exhibited reduced force (−40%, *p* < 0.0001 vs. Ctrls) and increased atrogene expression (3‐fold, *p* < 0.003 vs. Ctrls). Cancer altered white adipose tissue (WAT)'s functional gene signature (49%, *p* < 0.05 vs. Ctrls), whereas housing temperature reduced brown adipose tissue (BAT)'s (−78%, *p* < 0.05 vs. ST Ctrl). Thermogenic capacity measured by *Ucp1* expression decreased upon cancer in both WAT and BAT (−93% and −63%, *p* < 0.0044 vs. Ctrls). Cancer‐driven glucose intolerance was noted at ST (26%, *p* = 0.0192 vs. ST Ctrl), but restored at TN (−23%, *p* = 0.005 vs. ST C26). Circulating FGF21, GDF‐15 and IL‐6 increased in all C26 mice (4‐fold, *p* < 0.009 vs. Ctrls), with a greater effect on IL‐6 at TN (76%, *p* = 0.0018 vs. ST C26). Tumour and WAT *Il6* mRNA levels remained unchanged, while cancer induced skeletal muscle (SkM) *Il6* (2‐fold, *p* = 0.0016 vs. Ctrls) at both temperatures. BAT *Il6* was only induced in C26 mice at TN (116%, *p* = 0.0087 vs. ST C26). At the bioenergetics level, cancer increased SkM SERCA ATPase activity at ST (4‐fold, *p* = 0.0108 vs. ST Ctrl) but not at TN. In BAT, O_2_ consumption enhanced in C26 mice at ST (119%, *p* < 0.03 vs. ST Ctrl) but was blunted at TN (−44%, *p* < 0.0001 vs. ST C26). Cancer increased BAT ATP levels regardless of temperature (2‐fold, *p* = 0.0046 vs. Ctrls), while SERCA ATPase activity remained unchanged at ST and decreased at TN (−59%, *p* = 0.0213 vs. TN Ctrl).

**Conclusions:**

In mild cachexia, BAT and SkM bioenergetics are susceptible to different housing temperatures, which influences cancer‐induced alterations in glucose metabolism and systemic responses.

## 1 Introduction

Cancer cachexia (CC) is a metabolic condition affecting up to 80% of patients with cancer [1], mediated by reduced skeletal muscle (SkM) and fat mass and impaired function [2]. It dramatically compromises the quality of life, increases treatment toxicity and lowers survival rate. Despite exciting recent discoveries obtained using mouse models that describe potentially targetable molecular mechanisms, the enthusiasm is tempered by their failure to fully replicate the human condition [3].

Housing laboratory mice at ambient or standard temperature (ST; 20°C–22°C) results in metabolic adaptations driven by cold stress‐induced thermogenic activity, which do not occur when housing mice at their thermoneutral zone (TN; 28°C–32°C) [4]. Chronic cold stress elevates the sympathetic drive, leading to activated thermogenesis, increased heart and pulmonary rates and reduced immune suppression [4]. These effects contribute to an exacerbated energy expenditure and basal metabolic rate, resulting in adverse effects on overall metabolic health. In fact, we and others have shown that, compared to TN housing, ST housing affects metabolic adaptations in preclinical models of obesity [5], insulin action [6] and in response to exercise [7, 8], showing that housing mice under cold stress represents a substantial confounding factor. Although CC is considered a metabolic condition, the impact of housing temperature on murine CC remains unexplored. Thus, in this study, we assessed the contribution of cold‐induced thermogenic activity to the overall murine CC phenotype by housing tumour‐bearing mice at ST or TN conditions and analysing the whole‐body and tissue‐specific molecular responses to cancer.

We aimed to determine whether housing tumour‐bearing mice at standard temperature or thermoneutral conditions results in disparate whole‐body and molecular responses to cancer. We show that key systemic and intracellular signatures induced by cancer critically depend on housing temperature. These include glucose tolerance, IL‐6‐mediated systemic inflammation and intracellular adaptations related to bioenergetics of thermogenic tissues. Thus, we conclude that housing temperature impacts the cancer‐induced responses differently depending on the thermogenic tissue, and we propose that, under thermoneutral conditions, alterations in brown adipose tissue are major contributors to the overall CC phenotype.

## 2 Materials and Methods

### 2.1 Animals

Fourteen‐week‐old male BALB/C mice (Janvier) were kept on a 12:12‐h light–dark cycle with nesting material and ad libitum access to standard rodent chow diet (altromin no. 1324; Chr. Pedersen, Denmark). Mice were randomly assigned to standard temperature (22°C ± 1°C) or thermoneutrality (30°C ± 1°C). After a 3‐week acclimation period, mice were housed in pairs or triplets. Colon26 (C26) adenocarcinoma cells (Cytion, #400156) were cultured in RPMI 1640 medium (Gibco, #11875093) with 10% FBS (Sigma‐Aldrich, #F0804) and 1% penicillin–streptomycin (ThermoFisher, #15140122) at 37°C, 5% CO2. Before inoculation, C26 cells were trypsinized, washed with PBS and suspended in PBS at 5 × 10^6^ cells/mL. Mice were shaved on the right flank 1 day before subcutaneous injection of PBS with or without 5 × 10^5^ C26 cells. Body weight and composition were monitored weekly for the first 14‐days postinoculation, then twice weekly. Food intake was monitored twice weekly for the first 2 weeks and every other day during the last 4 to 7 days of intervention. Food shredding was not considered when analysing food intake. Mice with ulcerations > 5 mm or tumours > 14 mm (humane endpoint) were euthanized by cervical dislocation. Mice with tumours < 0.5 g were excluded. All experiments were approved by the Danish Animal Experimental Inspectorate (Licence: 2021‐15‐0201‐01085).

### 2.2 Body Composition

Total, fat and lean body mass were measured weekly by nuclear magnetic resonance using a Bruker LF90II Body Composition Analyser (Bruker). Body composition and weight were measured in all mice immediately prior to their dissection, and the final lean mass of C26 mice resulted from the subtraction of the tumour weight to the lean mass recorded prior dissection.

### 2.3 Grip Strength Assessment

Grip strength was assessed 2 days prior to the dissection. Mice were placed on the grip meter (Bioseb) with their forelimbs on the grid. Once on the apparatus, traction was applied via the tail. The resistance the mouse applied to the grid was recorded, and each mouse had five runs.

### 2.4 Ex Vivo Force Assessment

*Soleus* muscles were carefully dissected and incubated at 30°C in oxygenated Krebs‐Ringer buffer (117‐mM NaCl, 4.7‐mM KCl, 2.5‐mM CaCl2, 1.2‐mM KH2PO4, 1.2‐mM MgSO4, 24.6‐mM NaHCO3, 0.1% bovine serum albumin and 5‐mM glucose) in a myograph system (820MS DT, Denmark) [9, 10]. Optimal muscle length was determined by maximal isometric twitch response (14 V, 0.5‐ms pulse width). Single twitch assessment (three 14 V, 0.5‐ms pulses > 30 s apart) provided maximal twitch force, time to peak force and half‐relaxation time. Tetanic contractions (14 V, 0.2‐ms pulse width, 6.50‐ms interval, 150 Hz, 75 pulses per train, 500‐ms train duration, 5000‐ms pause and 110 trains total) were analysed by area under the curve to assess total force production. Absolute force was normalized to muscle cross‐sectional area to determine specific force (muscle mass divided by muscle density [1.06 g/cm^3^], optimal length and fibre length coefficient [0.71 for soleus] [11]).

### 2.5 Glucose Tolerance Test

Glucose (Sigma‐Aldrich, #1083421000) was dissolved in saline at a concentration of 0.2 g/mL and intraperitoneally injected into 4‐h‐fasted mice (fasting single‐housed from 7:00 AM) at a dose of 2‐g/kg body weight. At time points 0, 20, 40, 60 and 90 min, blood glucose was measured using a glucometer (Bayer Contour; Bayer, Münchenbuchsee, Switzerland). At time points 0 and 20, blood was collected from the tail using EDTA‐containing collection tubes (Microvette, VWR).

### 2.6 Lipidomics in Plasma Samples

Lipid species quantification in plasma samples was performed as described by Shashikadze et al. [12]. After adding 25 μL of a mix of 77 deuterated internal standards, plasma samples were extracted twice using water, methanol and MTBE. The combined organic phases were evaporated with N2 and reconstituted in 275 μL running solvent (10‐mM ammonium acetate in dichloromethane [50:50, v/v]). Seventy‐five microliters was injected into a SCIEX Exion UHPLC‐system coupled to a SCIEX QTRAP 6500+ mass spectrometer with a SelexION differential ion mobility interface. The MRM‐based quantification method details are in Shashikadze et al. 2023 [12]. The raw dataset contained 989 lipid species, preprocessed using R (version 4.2.1). Quality control steps included removing lipids with > 35% missing values in pool samples (*n* = 114), group‐specific missingness of 50% (*n* = 1), coefficient of variation > 25% in QC‐pool samples (*n* = 32) and dispersion ratio > 50% (*n* = 65). After QC [13], 777 lipid species remained, with 319 missing values (1.4% of the dataset). Missing values were imputed using the k‐nearest‐neighbour approach with k = 10.

### 2.7 Plasma Measurements

Plasma levels of circulating cytokines and hormones were measured using a customized multiplex assay kit by V‐PLEX platform (Mesoscale, Maryland, USA) and following the manufacturer's instructions. In this kit, we included the cytokines IL‐6, TNF‐α, IFN‐γ and IL‐1β, and the hormones FGF21, insulin and leptin. Plasma GDF‐15 was measured using the Mouse/Rat GDF‐15 Quantikine ELISA Kit (R&D Systems, MGD150).

### 2.8 Respirometry

Interscapular BAT and *quadriceps* muscles were placed in ice‐cold BIOPS solution (OROBOROS Instruments, Innsbruck, Austria) immediately after dissection. BAT was cut into small pieces, and muscle fibres were mechanically separated with needles. Tissues were then permeabilized with 2.5‐μg/mL digitonin for BAT and 50‐μg/mL saponin for muscle fibres for 30 min on ice with shaking, followed by two 10 min washes in MIRO5 medium (OROBOROS Instruments, Innsbruck, Austria). Approximately 2–3 mg of muscle fibres or BAT were transferred to the oxygraph‐2k (OROBOROS Instruments, Innsbruck, Austria) containing respiration medium. Respiration measurements were performed at 37°C in hyperoxia (O_2_: 200–450 μmol/L). The protocol included adding 10‐mM glutamate, 2‐mM malate and 5‐mM sodium pyruvate to assess resting respiration (State II), followed by 2.5‐mM ADP for State III. Uncoupling was evaluated by stepwise addition of 1‐μM CCCP until the optimum concentration was reached. Complexes I and III were inhibited by adding 0.5‐μM rotenone and 2.5‐μM antimycin A to evaluate non‐mitochondrial respiration.

### 2.9 Tissue Processing

All tissues were quickly dissected and snap frozen in liquid nitrogen. BAT and skeletal muscles were pulverized using a Bessman type tissue pulverizer (Cellcrusher kit) on dry ice. For RNA extraction, tissues were homogenized in QIAzol lysis reagent (#79306, Qiagen) and three sterile ceramic beads. For ATP determination, tissues were homogenized in 150 μL of ATP Assay Buffer from the ATP Colorimetric/Fluorimetric Assay Kit (Sigma‐Aldrich, MAK190). For protein extraction, tissues were homogenized in ice‐cold homogenization buffer (10% glycerol, 1% NP‐40, 20‐mM sodium pyrophosphate, 150‐mM NaCl, 50‐mM HEPES [pH 7.5], 20‐mM β‐glycerophosphate, 10‐mM NaF, 2‐mM phenylmethylsulfonyl fluoride [PMSF], 1‐mM EDTA [pH 8.0], 2‐mM Na_3_VO_4_, 10‐μg/mL leupeptin, 10‐μg/mL aproptinin and 3‐mM benzamidine) with a steel bead using the TissueLyser II bead mill (Qiagen, USA) for 1 min at 30 Hz.

### 2.10 Gene Expression Analyses

RNA was extracted using the RNeasy Mini Kit (Qiagen, #74106) per the manufacturer's instructions. RNA purity was assessed with the Nanodrop 2000/20000c spectrometer and ND1000 software (ThermoFisher Scientific). Reverse transcription was performed with 4 μL of qScript Ultra SuperMix (#95217, Quantabio) per 500–1000 ng of RNA. Gene expression was quantified by real‐time quantitative PCR using SYBR green (Applied Biosystems) and the QuantStudio 6 and 7 Real‐Time PCR System (Applied Biosystems), following the default comparative Ct protocol. Measurements were normalized to housekeeping genes (*b‐actin* or *36b4*). Primers and their sources are listed in Table S1.

### 2.11 ATP Determination

For the ATP determination assay, the deproteinizing sample preparation kit—TCA (Abcam, ab204708) was used on pulverized BAT and *triceps* muscles to remove interfering macromolecules. Homogenized tissues were incubated with 15 μL of ice‐cold TCA on ice for 15 min, then centrifuged at 12000 ×*g* for 5 min at 4°C. Supernatants were collected, and excess TCA was neutralized with 10 μL of cold neutralization solution. Samples were vented for 5 min on ice to release CO_2_ and stored at −80°C until ATP determination. The ATP Assay Kit (Sigma‐Aldrich, #MAK190) was used to measure fluorescence per the manufacturer's instructions.

### 2.12 Immunoblotting

After tissue homogenization for protein extraction, samples were rotated end‐over‐end for 30 min at 4°C, then centrifuged at 9500 ×*g* for 20 min at 4°C. Supernatants were collected and stored at −80 C. These steps were repeated three times for BAT to avoid fatty acid contamination. Protein concentration was measured using the BCA method (ThermoFisher, #23225) with BSA as the standard (Sigma‐Aldrich, #P0834). Twenty micrograms of protein extracts were resolved in 7%, 10% or 12.5% Mini‐PROTEAN precast acrylamide gels (BioRad) for SDS‐PAGE, transferred to PVDF membranes and blocked in TBS‐Tween 20 with 2% milk protein for 30 min at room temperature. Membranes were incubated with primary antibodies overnight at 4°C, followed by HRP‐conjugated secondary antibodies for 60 min at room temperature. Pierce Reversible Stain (ThermoFisher, #24585) or Coomassie Brilliant Blue R‐250 (Bio‐Rad, #1610400) was used as loading control. Bands were visualized using the Bio‐Rad ChemiDoc MP Imaging System and ECL+ (Amersham Biosciences). Band densitometry was performed with Quantity One (Bio‐Rad). Primary antibodies are listed in Table S2.

### 2.13 SERCA ATPase Activity Assay

To determine SERCA ATPase activity, an enzyme‐linked spectrophotometric assay was used, as previously described [14, 15, 16], in the absence of ionophore (i.e., presence of Ca^2+^ gradient). Briefly, BAT or *triceps* muscle homogenates were added to a reaction buffer with maximally activating Ca^2+^ concentrations (*p*Ca 5) and plated in duplicate in a 96‐well plate. The reaction was initiated with the addition of NADH. The rate of NADH disappearance, which occurs in a 1:1 ratio with ATP hydrolysis, was measured over 30 min. A baseline rate was obtained using the SERCA‐specific inhibitor, cyclopiazonic acid, which was subtracted from all other rates to obtain SERCA‐specific ATPase activity. SERCA activity was then normalized to sample protein concentration.

### 2.14 Statistics and Graphics

Data were collected using Excel 2016 and analysed with GraphPad Prism 10.1.1 for Windows (GraphPad Software). Data are expressed as mean ± SE, including individual values where applicable. Significance was set at α = 0.05. Student's T‐test was used to compare one variable between two groups. Two‐way ANOVA with Tukey's post‐hoc test was used for comparing one or multiple variables across four groups. Data plots were generated with GraphPad Prism 10.1.1, and graphics in Figure 5 were created with BioRender (biorender.com).

## 3 Results

Housing temperature does not affect cancer‐induced effects on body weight and composition, muscle force and atrophy, but influences the brown adipose tissue gene signature.

**FIGURE 1:**
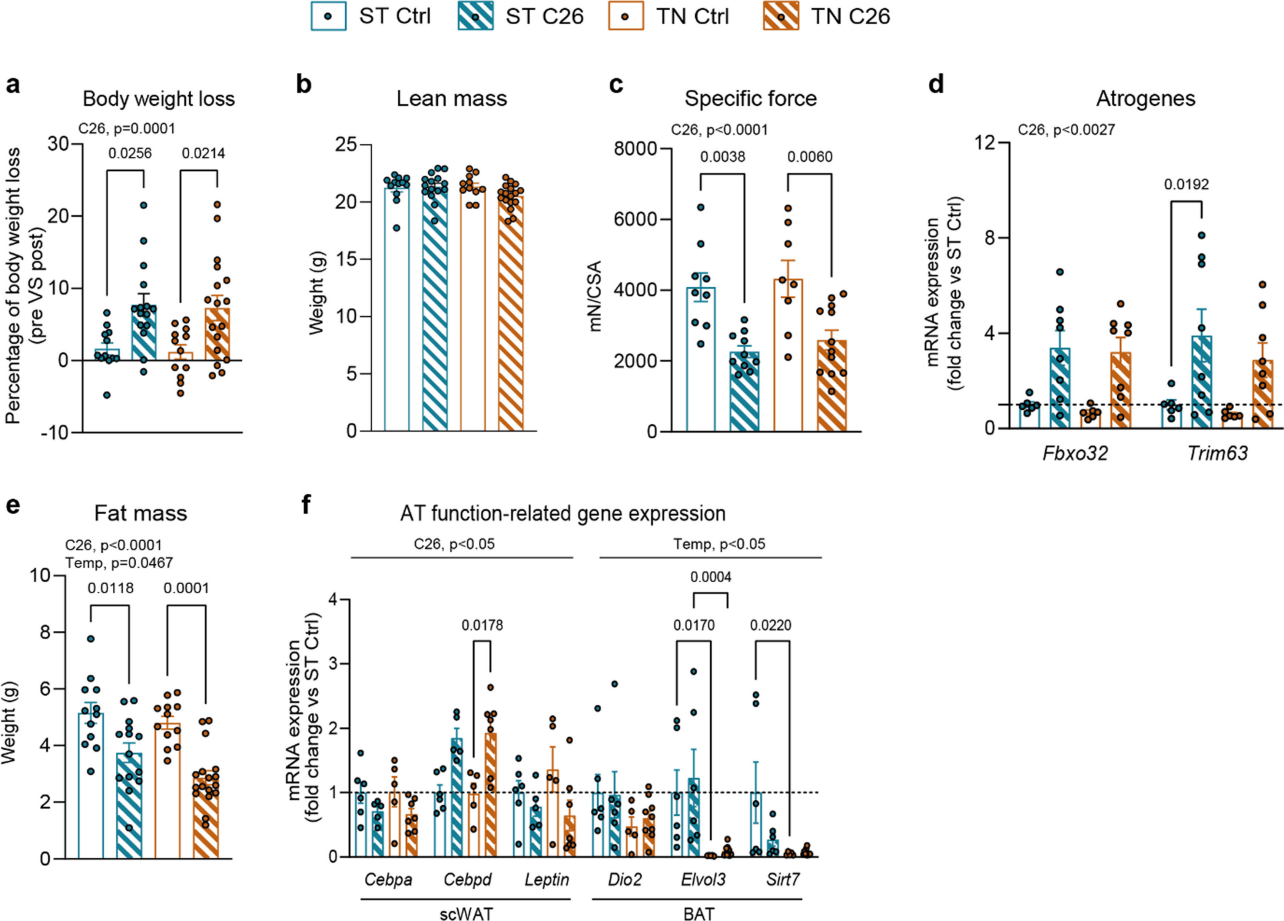
Housing temperature does not affect body weight and composition, muscle force and atrophy but influences the brown adipose tissue (BAT) gene signature upon cancer. (a) Percentage of tumour‐free body weight loss comparing the body weight right before C26 cell inoculation (pre) and right before dissection (post). (b) Tumour‐free lean mass. (c) Specific force in soleus muscles. (d) *Fbxo32* and *Trim63* atrogene expression in SkM. (e) Fat mass. (f) WAT and BAT‐related gene expression. Data are expressed as mean ± SE including individual values where applicable. (a–f) Two‐way ANOVA test with Tukey's post hoc test. C26, main cancer effect; Temp, main temperature effect.

To first establish the effect of housing temperature on classical CC manifestations, we determined body weight and composition, food intake, SkM and fat mass and function in control (Ctrl) or C26 tumour‐bearing mice housed at standard temperature (ST; 20°C–22°C) or thermoneutral temperature (TN; 28°C–30°C). Eighteen to twenty‐one days after subcutaneous inoculation of C26‐colon carcinoma cells, we observed an average 7.5% body weight loss (see Figure 1a), along with increased spleen mass (see Figure S1a), in all C26 mice irrespective of housing temperature. Food intake (Figure S1b), tumour (Figure S1c) and liver weight (Figure S1d) remained unchanged. This corresponds to mild cachexia as previously defined in mice with body weight loss up to 10% [17].

CC is characterized by reduced SkM mass and function. In this regard, lean mass (Figure 1b) and SkM weight (Figure S1e) remained unaltered. Despite no significant reduction in grip strength (Figure S1f), ex vivo force assessment showed substantially decreased force in C26 mice independently of temperature (−42%; Figure 1c). We also found upregulated atrophy‐related gene (atrogene) expression (Figure 1d), indicating that SkM weakness and atrophy precede loss of SkM mass upon cancer irrespective of housing temperature. As previously reported [7], TN lowered heart weight (Figure S1g), while only cancer induced cardiac atrogene expression (Figure S1h).

CC is also characterized by alterations in fat mass and function [18]. Indeed, all C26 mice showed reduced total fat mass (Figure 1e) compared to their controls, explained by an average 24% decrease gonadal and subcutaneous white adipose tissue weight (gWAT and scWAT, respectively) (Figure S1i). The interscapular brown adipose tissue (iBAT, hereafter BAT) showed the same trend (Figure S1j). As a readout for adipose tissue function, we evaluated mRNA levels of key genes involved in WAT and BAT homeostasis. We analysed *Cebpa*, *Cebpd* (markers for late and early stage adipogenesis, respectively [19]) and *Leptin* (marker of fat storage [20]) in WAT. In BAT, we analysed *Dio2*, *Elvol3* and *Sirt7*, essential in driving cold‐induced thermogenesis [21]. We observed that cancer (main effect), and not temperature, promoted an mRNA expression profile indicative of altered adipogenic capacity in WAT (Figure 1f). As expected, the expression of thermogenic‐related genes in BAT was reduced under TN conditions (Figure 1f). Notably, the expression of thermogenic‐related genes in BAT was unchanged in C26 mice housed at ST, which notably contrasts with previous studies conducted at ST showing increased thermogenic activity in BAT during CC [18, 22]. To further assess the thermogenic capacity in these conditions, we assessed the expression levels of uncoupling protein 1 (Ucp1), the master regulator of nonshivering thermogenesis in adipose tissues [23]. Ucp1 uncouples ATP production from oxidative phosphorylation, a process that dissipates heat. Aligned with previous studies [24], C26 mice housed at ST displayed a 27%, 88% and 45% downregulation of Ucp1 mRNA levels in scWAT, gWAT and BAT, respectively. Interestingly, these reductions were enhanced at TN (Figure S1k). Concomitantly, C26 mice at ST showed no changes in BAT Ucp1 protein content, whereas TN housing promoted a reduction (−67%, Figure S1l). Thus, especially at TN, cancer resulted in whitening of adipose tissue, altering its thermogenic capacity, which contrasts with previous studies undertaken at ST showing induction of browning in CC [18, 22]. Altogether, our data indicate that housing temperature differently influences cancer‐dependent adaptations especially in BAT of C26 mice with mild cachexia, which potentially impacts the overall metabolic status.

### 3.1 Glucose Metabolism and Circulating Factors, but Not Plasma Lipid Composition, Are Influenced by Housing Temperature Upon Cancer

**FIGURE 2:**
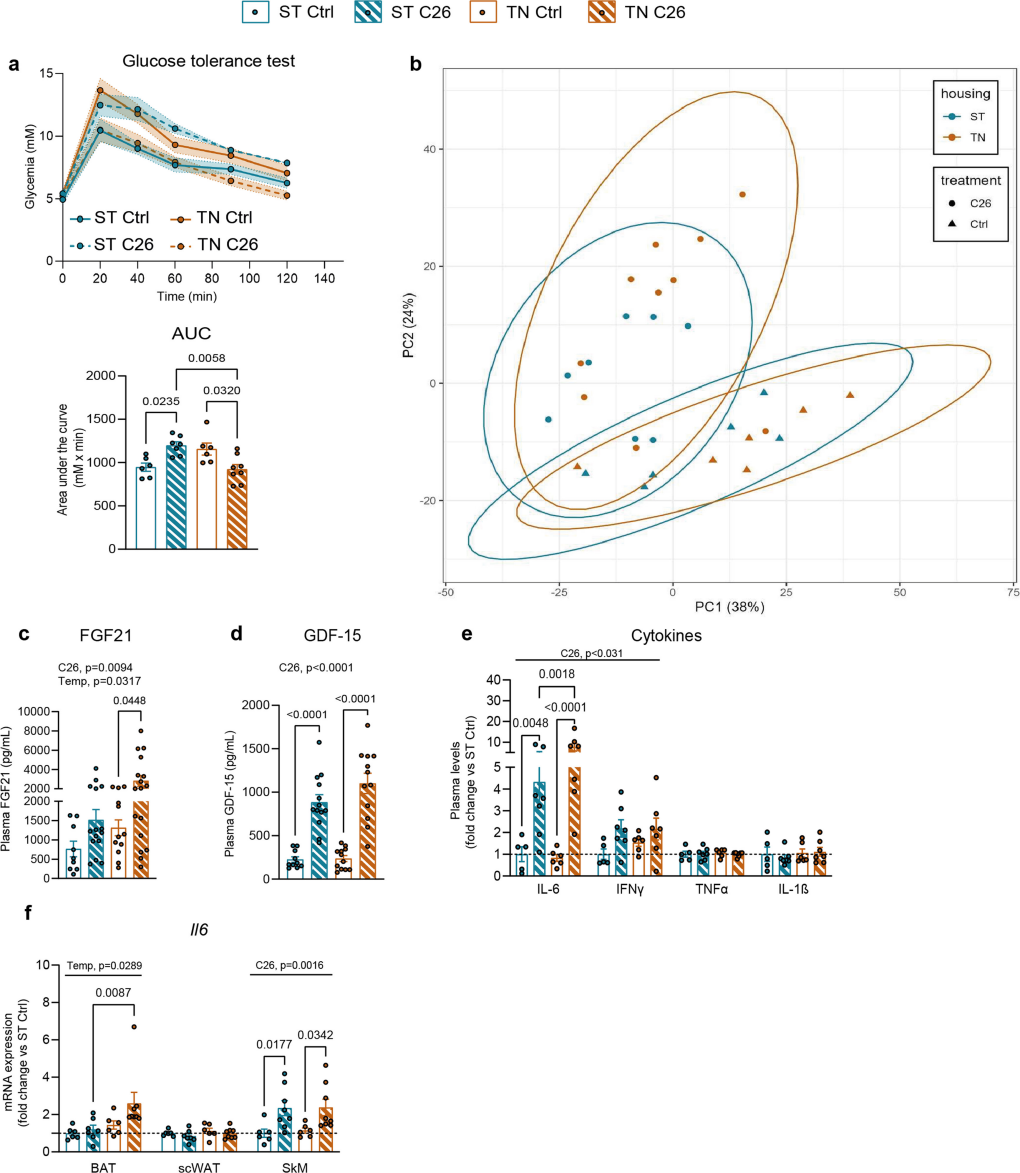
Glucose metabolism and circulating factors, but not plasma lipid composition, are influenced by housing temperature upon cancer. (a) Glycaemic response during a GTT and average area under the curve (ST control *n* = 6, ST C26 *n* = 7, TN control *n* = 6, and TN C26 *n* = 8). (b) Principal component analysis (PCA) scores plot of plasma lipid species concentrations. (c–e) Plasma levels of circulating factors. (f) IL‐6 gene expression. Data are expressed as mean ± SE including individual values where applicable. (a, c–f) Two‐way ANOVA with Tukey's post hoc test. C26, main cancer effect; Temp, main temperature effect.

Impaired glucose metabolism has been widely reported in response to cancer in mice [25, 26]. To assess glycemic control, we subjected the mice to a glucose tolerance test. Aligning with previous studies [25], C26 mice with mild cachexia housed at ST displayed glucose intolerance compared to controls (Figure 2a), along with a trend towards elevated fasting blood glucose and insulin (+17% and +77%, respectively) (Figure S2a,b). Strikingly, when housed at TN, C26 mice showed improved glucose tolerance (Figure 2a), unaltered fasting blood glucose and lowered fasting insulin (Figure S2a,b) compared to C26 mice at ST or control mice at TN. These data suggest a potential contribution of cold‐induced metabolism in thermogenic tissues to the adaptations in glycemic control upon cancer. Lipid metabolism is also strongly influenced by cancer [18, 27]. Therefore, we evaluated temperature‐dependent plasma lipid responses to cancer using lipidomics. Principal component analyses (PCA) of plasma showed clustering of cancer‐dependent lipid composition (Figure 2b). Recapitulating previous studies [27], all C26 mice showed enrichment in circulating modified ceramides, phenocopying human CC (Figure S2c). However, the effect of cancer on lipid metabolism seemed independent of housing temperature.

Both metabolic dysfunction and cancer are characterized by changes in circulating factors, such as fibroblast growth factor 21 (FGF21 [28]), growth differentiating factor 15 (GDF‐15 [29]), leptin [20] and inflammatory cytokines [30]. C26 mice exhibited an average 4‐fold increase in plasma FGF21 (Figure 2c) and GDF‐15 (Figure 2d) levels compared to their respective control mice, and this effect was augmented for FGF21 at TN by 86% compared to ST. Circulating leptin levels were reduced in all C26 mice independently of housing temperature (Figure S2d). We also found that C26 mice presented systemic inflammation, indicated by increased circulating IFN‐γ and IL‐6 (Figure 2e). Interestingly, circulating IL‐6 levels were 76% elevated in C26 mice housed at TN compared to ST. Thermogenic tissues are major sources of IL‐6; thus, we hypothesized that changes in housing temperature might affect the cancer‐induced IL‐6 production in such tissues. Our data demonstrated that SkM, but not adipose tissues, likely contributed to the IL‐6‐mediated inflammatory response in C26 mice at ST, whereas at TN, both BAT and SkM produced this cytokine (Figure 2f). These changes occurred concomitant with unaltered tumour *Il6* levels in C26 mice housed at ST or TN (Figure S2e). This indicates that housing temperature differentially influences the IL‐6 response to cancer from thermogenic tissues, especially in BAT.

### 3.2 Housing Temperature Has a Mild Impact on Cancer‐Induced SkM Bioenergetic Adaptations

**FIGURE 3:**
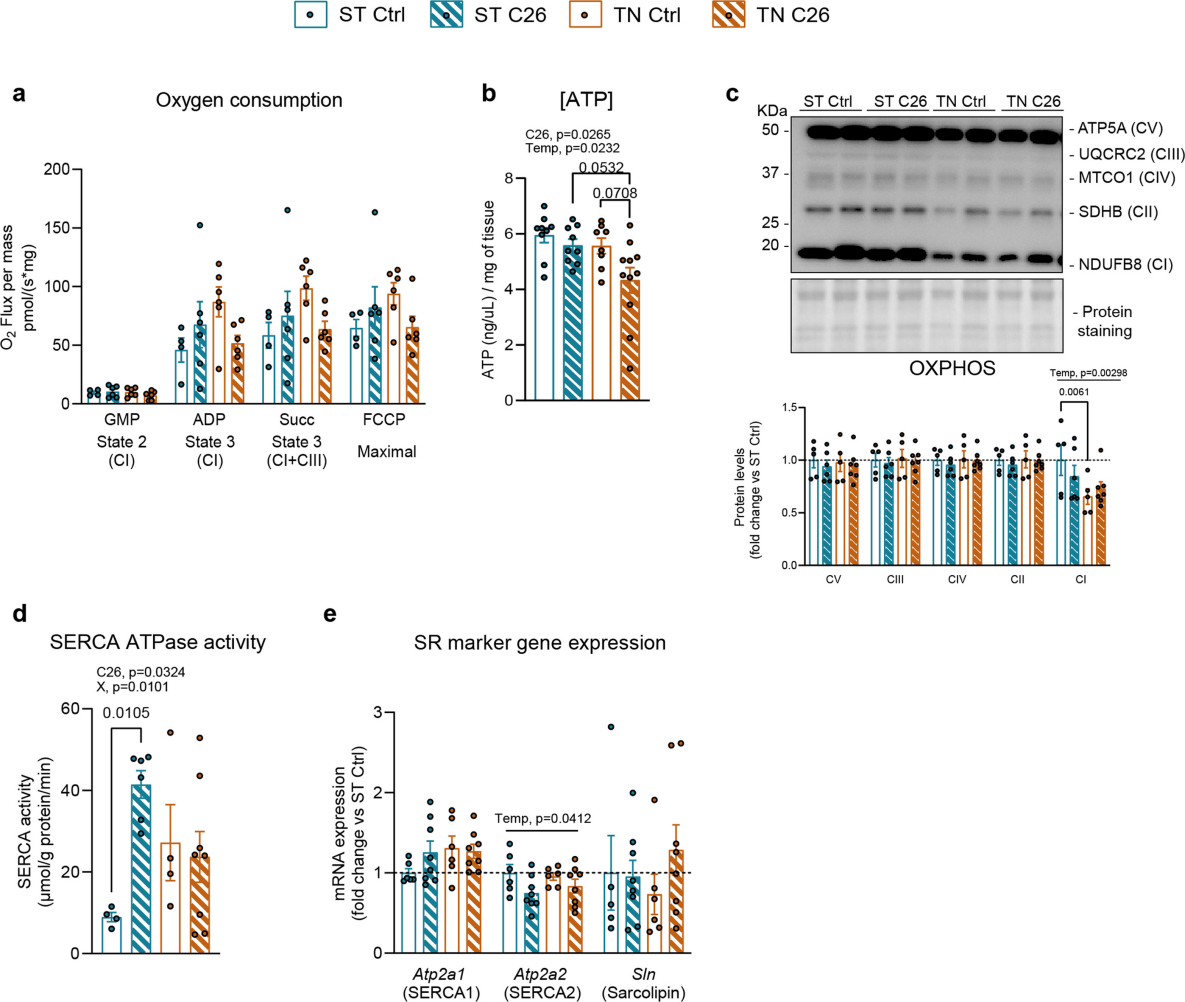
Housing temperature impacts cancer‐induced skeletal muscle bioenergetic adaptations. (a) Oxygen consumption in SkM fibres. (b) ATP concentration. (c) OXPHOS subunit representative immunoblots and quantification. (d) SERCA ATPase activity. (e) mRNA levels of SR markers. Data are expressed as mean ± SE including individual values where applicable. (a–e) Two‐way ANOVA test with Tukey's post hoc test. C26, main cancer effect; Temp, main temperature effect.

Cellular bioenergetics govern the thermogenic responses to cold stress in thermogenic tissues, and alterations in these processes have the potential to affect the overall metabolic status. Thus, we first evaluated the impact of ST or TN housing on molecular processes involved in cellular bioenergetics in SkM. First, as a functional readout of mitochondrial respiratory capacity, we evaluated O_2_ consumption in permeabilized SkM fibres. In C26 mice housed at ST, we observed a tendency for an increased O_2_ consumption in SkM fibres compared to controls (Figure 3a). Yet, in mice housed at TN, cancer seemed to lower SkM O_2_ consumption compared to controls. To investigate the outcome of these slight changes in O_2_ consumption, we measured SkM ATP levels. SkM ATP levels were largely unaltered, except for a trend towards a decrease in C26 mice housed at TN (Figure 3b). These alterations were independent of changes in mitochondrial mass, measured as TOMM20 protein content (Figure S3a) and mRNA expression of mitochondrial biogenesis markers *Tfam* and *Ppargc1a* (PGC1α) (Figure S3b). Furthermore, protein quantification of different subunits of the OXPHOS complex showed no changes upon cancer or temperature (Figure 3c), with the only exception of Complex I (CI), which was 34% lowered by TN. To further understand the housing temperature‐dependent effects on ATP levels upon cancer in SkM, we evaluated the activity of the sarco/endoplasmic reticulum Ca^2+^ ATPase (SERCA ATPase), which pumps Ca^2+^ ions from the cytosol to the sarco/endoplasmic reticulum (SR/ER). This requires ATP hydrolysis, and its uncoupling via sarcolipin results in heat dissipation, a key mechanism in nonshivering thermogenesis in SkM [31]. At ST housing, SkM SERCA ATPase activity was increased 4‐fold in C26 mice, while at TN, cancer did not influence SkM SERCA ATPase activity (Figure 3d). These changes occurred independently of mRNA levels of SR markers SERCA1, SERCA2 and Sarcolipin (Figure 3e), and protein levels of SERCA1 and SERCA2 (Figure S3c). Notably, we observed a TN‐induced reduction in protein levels of STIM1, an SR/ER‐residing Ca^2+^ sensor (Figure S3c). Reduced STIM1 levels have been associated with decreased mitochondria‐free Ca^2+^ and respiration [32], which suggests that the underlying molecular systems leading to increased SERCA ATPase activity upon cancer or changes in housing temperature are different.

### 3.3 The Cancer‐Induced Bioenergetic Responses in BAT Are Strongly Influenced by Housing Temperature

**FIGURE 4:**
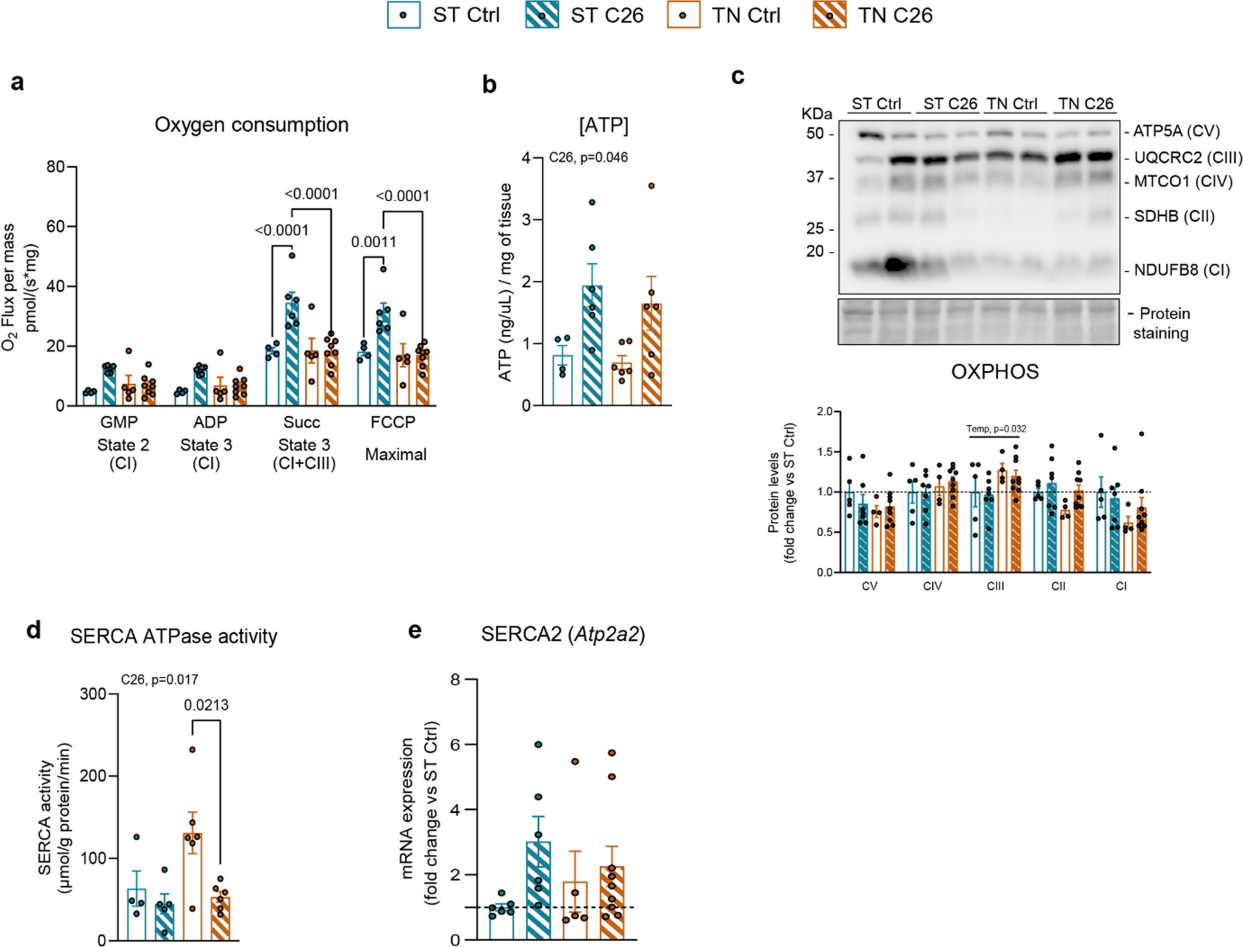
The cancer‐induced bioenergetic responses in brown adipose tissue are strongly influenced by housing temperature. (a) Oxygen consumption in permeabilized BAT. (b) ATP concentration. (c) OXPHOS subunit representative immunoblots and quantification. (d) SERCA ATPase activity. (e) SERCA2 mRNA levels. Data are expressed as mean ± SE including individual values where applicable. (a–e) Two‐way ANOVA test with Tukey's post hoc test. C26, main cancer effect; Temp, main temperature effect; X, interaction between cancer and temperature.

Next, we evaluated the cellular bioenergetics of BAT. We observed a 2‐fold increase in O_2_ consumption in permeabilized BAT of C26 mice housed at ST, which was completely blunted at TN (Figure 4a). Yet, all C26 mice presented increased BAT ATP levels (Figure 4b). These adaptations were not due to alterations in mitochondrial mass, with only a modest increase in TOMM20 protein upon cancer regardless of temperature (+15%, Figure S4a), accompanied by a decreasing trend of *Tfam* and *Ppargc1a* (Figure S4b). Similar to SkM, BAT protein levels of OXPHOS subunits remained unaltered in all C26 mice, with the exception of CIII, which was increased by temperature (Figure 4c). These data indicate that bioenergetics of BAT display markedly different cancer‐induced phenotypes depending on housing temperature. To evaluate the biology of ATP levels in BAT and Ucp1‐independent nonshivering thermogenesis, we also assessed SERCA ATPase activity in this tissue. Unlike in SkM, BAT of C26 mice housed at ST showed a 29% reduction in SERCA ATPase activity compared to controls, and this reduction was enhanced at TN (Figure 4d). Indeed, the exacerbated reduction observed at TN was due to the fact that control mice at TN exhibited a marked increase compared to controls at ST. Thus, TN housing impacts BAT SERCA ATPase activity, an effect diminished by cancer. This may explain why BAT of C26 mice housed at TN exhibits elevated ATP levels without increased oxygen consumption through oxidative phosphorylation. Interestingly, SERCA2 mRNA levels tended to be increased in C26 mice housed at ST, but not at TN, when compared to controls (Figure 4e), although its protein levels in all C26 mice were reduced (−14%, Figure S4c). Unlike in SkM, STIM1 was unaltered upon cancer or temperature in BAT. These data indicate that the cancer‐induced effects on ATP regulation depend on housing temperature in BAT.

Altogether, our data demonstrate that housing temperature impacts metabolic and molecular responses to cancer, including alterations in glucose metabolism, and IL‐6‐driven inflammatory responses. We found that thermogenic tissues are differently affected by cancer or temperature, and BAT shows the most dramatic temperature‐dependent changes upon cancer. These observations set a precedent for the consideration of housing temperature as a contributing factor to the cancer‐induced responses in vivo models, and could explain, at least to some extent, the lack of translation from preclinical to clinical CC.

## 4 Discussion

Preclinical mouse models have significantly advanced our understanding of cancer's molecular and organismal impacts and are routinely used to test new drugs [33]. However, we show that housing temperature crucially affects responses to murine cancer, highlighting the need to consider this factor in study design.

A remarkable finding is that the bioenergetic adaptations to cancer occurring in BAT and SkM depended on housing temperature. We unprecedentedly show that these temperature‐dependent alterations include a 4‐fold increase in SkM SERCA ATPase activity accompanied by a 2‐fold increase in BAT O_2_ consumption in C26 mice housed at ST, but not at TN. Interestingly, we found unaltered Ucp1 protein content in BAT of C26 mice housed at ST, recapitulating previous observations made in WAT under similar conditions [17]. Thus, these data indicate that the cancer‐mediated adaptations in the mitochondrial respiratory capacity of BAT are sensitive to temperature; however, they do not correlate with Ucp1 content and, consequently, with thermogenic activity. In this regard, we observed decreased Ucp1 gene expression in BAT and WAT of mice with mild cachexia, recapitulating a previous study [24]. Therefore, these findings feed the debate of whether CC is driven by WAT browning and exacerbated energy expenditure [18, 22].

Importantly, our findings on increased SERCA ATPase activity in SkM of C26 mice housed at ST, and not at TN, also support the notion that, upon cancer, the increased demand for heat production at ST is met by SkM SERCA‐mediated thermogenesis, and not by Ucp1‐mediated thermogenesis in BAT. Thus, these observations further challenge the prevailing hypothesis that BAT and WAT browning are responsible for increased energy expenditure in CC. Intriguingly, we found unaltered protein content of SERCA and sarcolipin in conditions where SkM and BAT SERCA ATPase activity were induced. Previous studies have reported this observation and demonstrated that parameters, such as changes to the membrane lipid composition of the SR/ER [34]—among others—can alter SERCA energetics without changes in total protein content.

In this regard, altered SERCA ATPase activity can result in SkM atrophy [35]. Yet, SkM weakness and induction of atrogenes preceded SkM mass loss in C26 mice and were not influenced by housing temperature, indicating that increased SERCA ATPase activity may only contribute to muscle weakness upon cancer at ST. We also found that loss of fat mass upon cancer was not affected by housing temperature, although interestingly, while the expression of genes involved in WAT function was affected only by cancer, gene expression involved in BAT activity was only temperature‐dependent.

Another set of key findings were that glucose metabolism was considerably affected by temperature in C26 mice, which could be due to relevant circulating factor playing a role in glycemic control, such as FGF21 [36]. Interestingly, cancer increased plasma FGF21 levels, and this increase was enhanced upon TN. The same observation was made for the cytokine IL‐6, which has also been shown to improve glucose metabolism in both healthy and obese mice [37]. Thus, it could be speculated that C26 mice housed at TN present improved glucose metabolism due to the synergistic and enhanced effect of FGF21 and IL‐6. Yet, because of IL‐6's effects on SkM and adipose tissues, this also results in a detrimental impact further driving tissue dysfunction.

**FIGURE 5:**
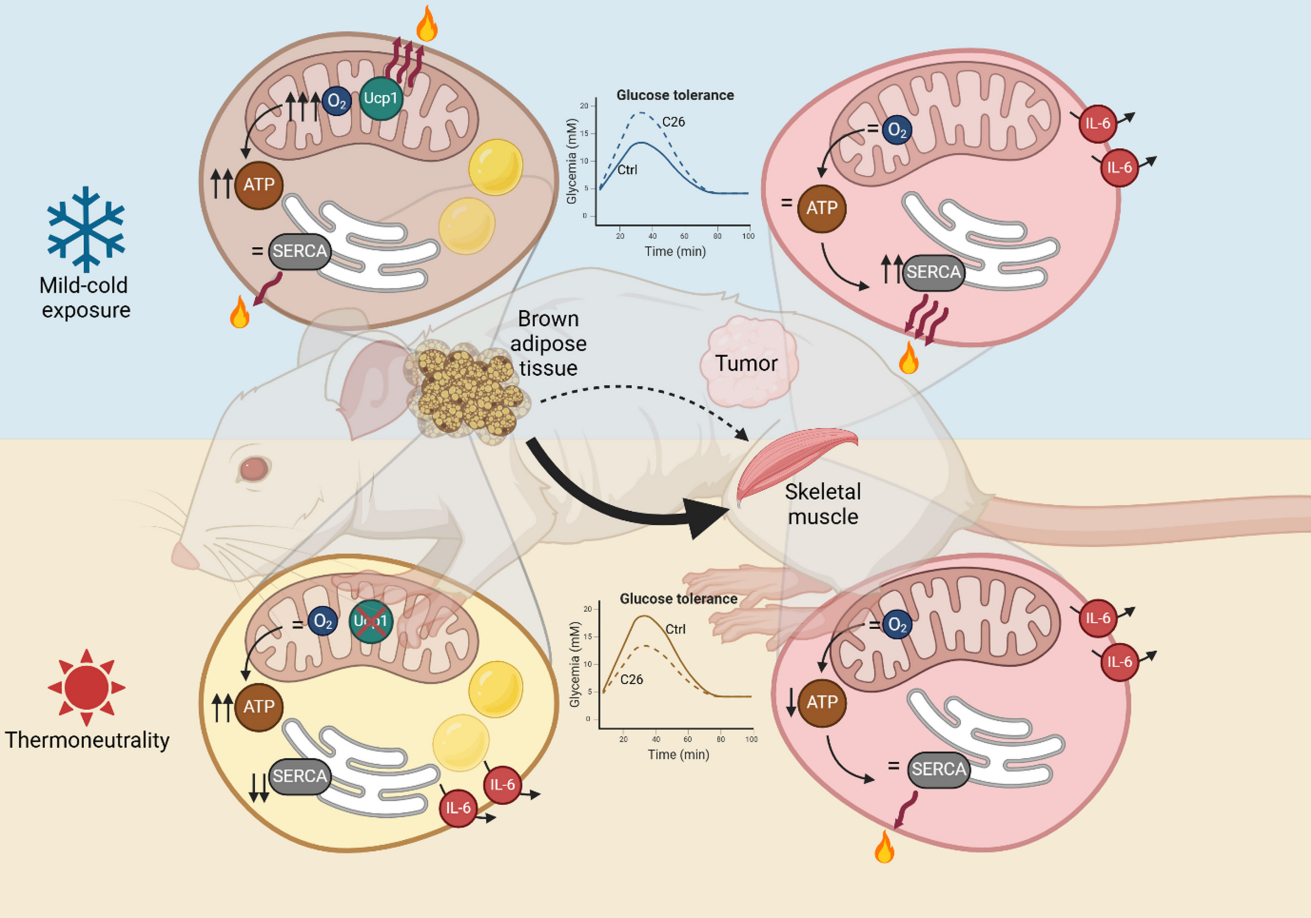
Housing temperature impacts the systemic and tissue‐specific molecular responses to cancer in mice. Graphical abstract depicting our findings on the key systemic and intracellular signatures induced by cancer that critically depend on housing temperature. These include adaptations in glucose tolerance, IL‐6‐mediated systemic inflammation and bioenergetics of thermogenic tissues, such as the BAT mitochondrial respiratory capacity and SkM SERCA ATPase activity.

Altogether, our data demonstrate that housing temperature impacts metabolic and molecular responses to cancer in mice, including alterations in body composition, glucose metabolism and inflammatory responses. Our study also shows that housing temperature is a determinant factor in the cancer‐induced cellular responses in mice, which are especially influenced in thermogenic tissues. Due to known interactions between BAT and other tissues in both basal and during cancer [38], we speculate that initial cancer‐triggered alterations in BAT bioenergetics could signal other tissues, especially SkM (Figure 5). Importantly, humans live in their TN zone, thereby not requiring thermogenesis from BAT [39]. Opposed to this notion, a recent study assessing both healthy subjects and patients with cancer, along with different tumour‐bearing mouse models, demonstrated that a significant amount of BAT exists in humans, that it is activated upon cold exposure and that cold‐induced metabolic activity of BAT compromises tumour glucose uptake [40]. Our study, along with existing evidence, highlights the significant role of cold‐induced BAT and SkM metabolism during cancer in both humans and mice, enhancing our understanding of translating preclinical findings to human pathophysiology.

## Author Contributions

Conceptualization: A.I and L.S. Methodology: A.I., T.C.P.P., J.B., A.M.E., M.H., C.H.F.H., R.M‐V, S.L. and L.S. Investigation: A.I., E.F., T.C.P.P., J.B., A.M.E., M.H., F.R., C.H.F.H., A‐S.R.J., N.R.A., L‐H‐C., R.M‐V., M.S.A., S.H.R., J.L.M., S.G., S.L., Z.G‐H., T.E.J., M.R., J.T.T., V.A.F. and L.S. Visualization: A.I. and L.S. Supervision: L.S. Writing (original draft): A.I. and L.S. Writing (review and editing): A.I., E.F., T.C.P.P., J.B., A.M.E., M.H., F.R., C.H.F.H., A‐S.R.J., N.R.A., L‐H‐C., R.M‐V., M.S.A., S.H.R., J.L.M., S.G., S.L., Z.G‐H., T.E.J., M.R., J.T.T., V.A.F. and L.S.

## Conflicts of Interest

The authors declare no conflicts of interest.

## Supporting information


**Figure S1** (a) Spleen weight relative to tumour‐free final body weight. (b) Average food intake per mouse and day over the last 4 to 7 days of intervention. (c) Tumour weight. (d) Liver weight relative to tumour‐free final body weight. (e) Muscle weights relative to tumour‐free final body weight. (f) Grip strength quantification. (g) Heart weight relative to tumour‐free final body weight. (h) Atrogene expression in heart. (i) Weights of WAT depots relative to tumour‐free final body weight. (j) iBAT weight related to tumour‐free final body weight. (k) Ucp1 gene expression. (l) Representative immunoblot of Ucp1 and band quantification in BAT. Data are expressed as mean ± SE including individual values where applicable. (a, c–l) Two‐way ANOVA with Tukey’s post‐hoc test. (b) Student’s T‐test. C26, main cancer effect; Temp, main temperature effect; X, interaction between cancer and temperature.
**Figure S2.** (a) Fasting blood glucose. (b) Fasting blood insulin. (c) Plasma lipid class sum concentration. (d) Plasma leptin levels. (e) IL‐6 gene expression in tumours. Data are expressed as mean ± SE including individual values where applicable. (a, b, d) Two‐way ANOVA test with Tukey’s post‐hoc test. (c) Wilcoxon test. (e) Student’s T‐test. C26, main cancer effect; Temp, main temperature effect.
**Figure S3.** (a) Representative immunoblot of TOM20 and band quantification in SkM. (b) *Tfam* and *Ppargc1a* mRNA levels SkM. (c) Representative immunoblots of SR markers and band quantification in SkM. Data are expressed as mean ± SE including individual values where applicable. (a–c) Two‐way ANOVA test with Tukey’s post‐hoc test. Temp, main temperature effect.
**Figure S4.** (b) Representative immunoblot of TOM20 and band quantification in BAT. (b) *Tfam* and *Ppargc1a* mRNA levels BAT. (c) Representative immunoblots of ER markers and band quantification in BAT. Data are expressed as mean ± SE including individual values where applicable. (a–c) Two‐way ANOVA test with Tukey’s post‐hoc test. Temp, main temperature effect.
**Table S1:** SYBR green mouse primers.
**Table S2:** Primary antibodies.
