# Supplementary material for: Housing Temperature Impacts the Systemic and Tissue‐Specific Molecular Responses to Cancer in Mice
Source: J Cachexia Sarcopenia Muscle. 2025 Apr 16;16(2):e13781. doi: 10.1002/jcsm.13781 (PMC12001421; doi:10.1002/jcsm.13781)
Supplement: Supplementary file 1 — Figure S1 (a) Spleen weight relative to tumour‐free final body weight. (b) Average food intake per mouse and day over the last 4 to 7 days of intervention. (c) Tumour weight. (d) Liver weight relative to tumour‐free final body weight. (e) Muscle weights relative to tumour‐free final body weight. (f) Grip strength quantification. (g) Heart weight relative to tumour‐free final body weight. (h) Atrogene expression in heart. (i) Weights of WAT depots relative to tumour‐free final body weight. (j) iBAT weight related to tumour‐free final body weight. (k) Ucp1 gene expression. (l) Representative immunoblot of Ucp1 and band quantification in BAT. Data are expressed as mean ± SE including individual values where applicable. (a, c–l) Two‐way ANOVA with Tukey’s post‐hoc test. (b) Student’s T‐test. C26, main cancer effect; Temp, main temperature effect; X, interaction between cancer and temperature. Figure S2. (a) Fasting blood glucose. (b) Fasting blood insulin. (c) Plasma lipid class sum concentration. (d) Plasma leptin levels. (e) IL‐6 gene expression in tumours. Data are expressed as mean ± SE including individual values where applicable. (a, b, d) Two‐way ANOVA test with Tukey’s post‐hoc test. (c) Wilcoxon test. (e) Student’s T‐test. C26, main cancer effect; Temp, main temperature effect. Figure S3. (a) Representative immunoblot of TOM20 and band quantification in SkM. (b) Tfam and Ppargc1a mRNA levels SkM. (c) Representative immunoblots of SR markers and band quantification in SkM. Data are expressed as mean ± SE including individual values where applicable. (a–c) Two‐way ANOVA test with Tukey’s post‐hoc test. Temp, main temperature effect. Figure S4. (b) Representative immunoblot of TOM20 and band quantification in BAT. (b) Tfam and Ppargc1a mRNA levels BAT. (c) Representative immunoblots of ER markers and band quantification in BAT. Data are expressed as mean ± SE including individual values where applicable. (a–c) Two‐way ANOVA test with Tukey’s post‐hoc test. Temp, m [file JCSM-16-e13781-s001.pdf]

SUPPLEMENTARY MATERIAL

Figures

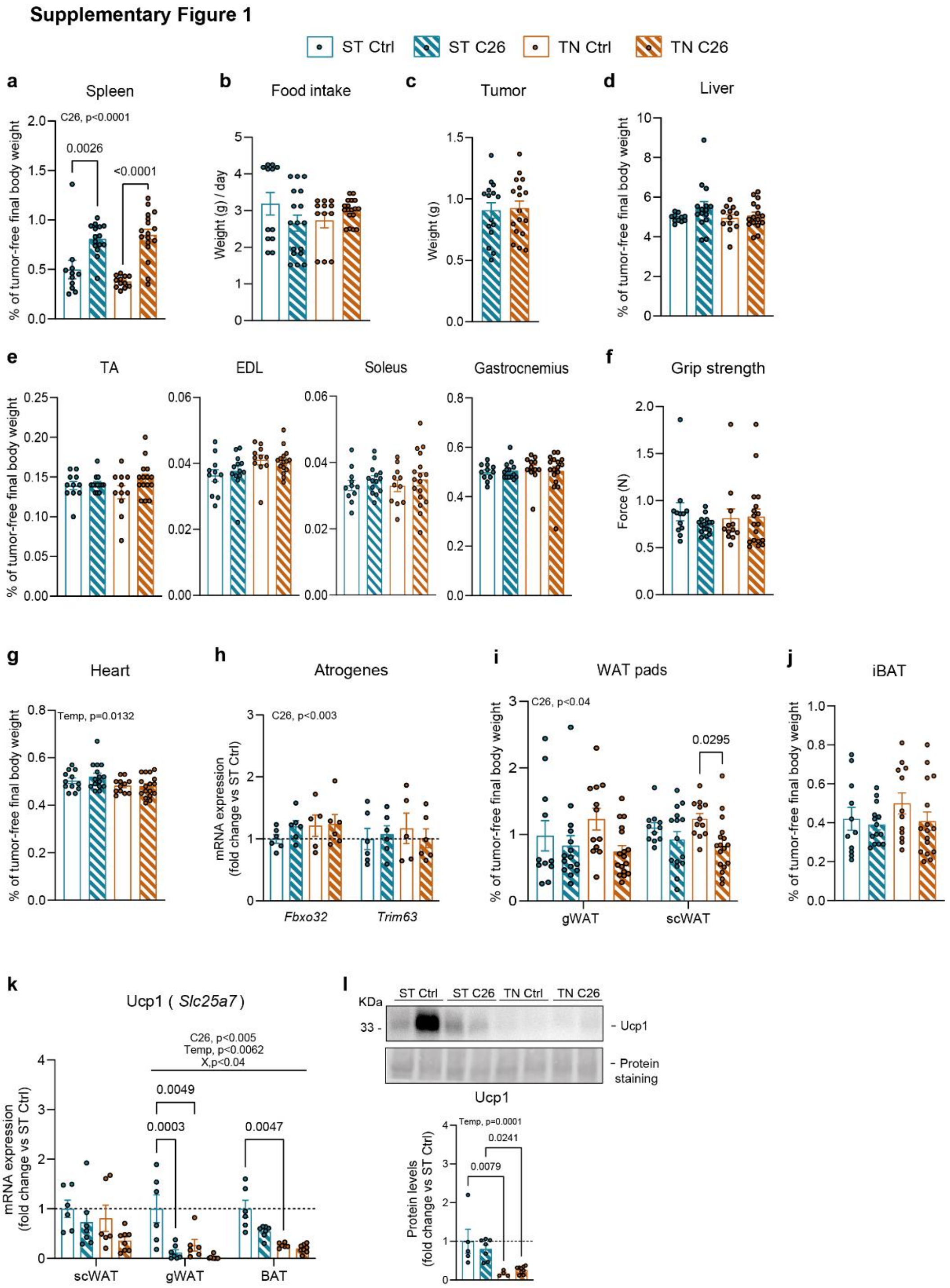

Supplementary Figure 1. (**a**) Spleen weight relative to tumor-free final body weight. (**b**) Average food intake per mouse and day over the last 4 to 7 days of intervention. (**c**) Tumor weight. (**d**) Liver weight relative to tumor-free final body weight. (**e**) Muscle weights relative to tumor-free final body weight. (**f**) Grip strength quantification. (**g**) Heart weight relative to tumor-free final body weight. (**h**) Atrogene expression in heart. (**i**) Weights of WAT depots relative to tumor-free final body weight. (**j**) iBAT weight related to tumor-free final body weight. (**k**) Ucp1 gene expression. (**l**) Representative immunoblot of Ucp1 and band quantification in BAT. Data are expressed as mean ± SE including individual values where applicable. (**a, c-l**) Two-way ANOVA with Tukey's post-hoc test. (**b**) Student's T test. C26, main cancer effect; Temp, main temperature effect; X, interaction between cancer and temperature.

Supplementary Figure 2

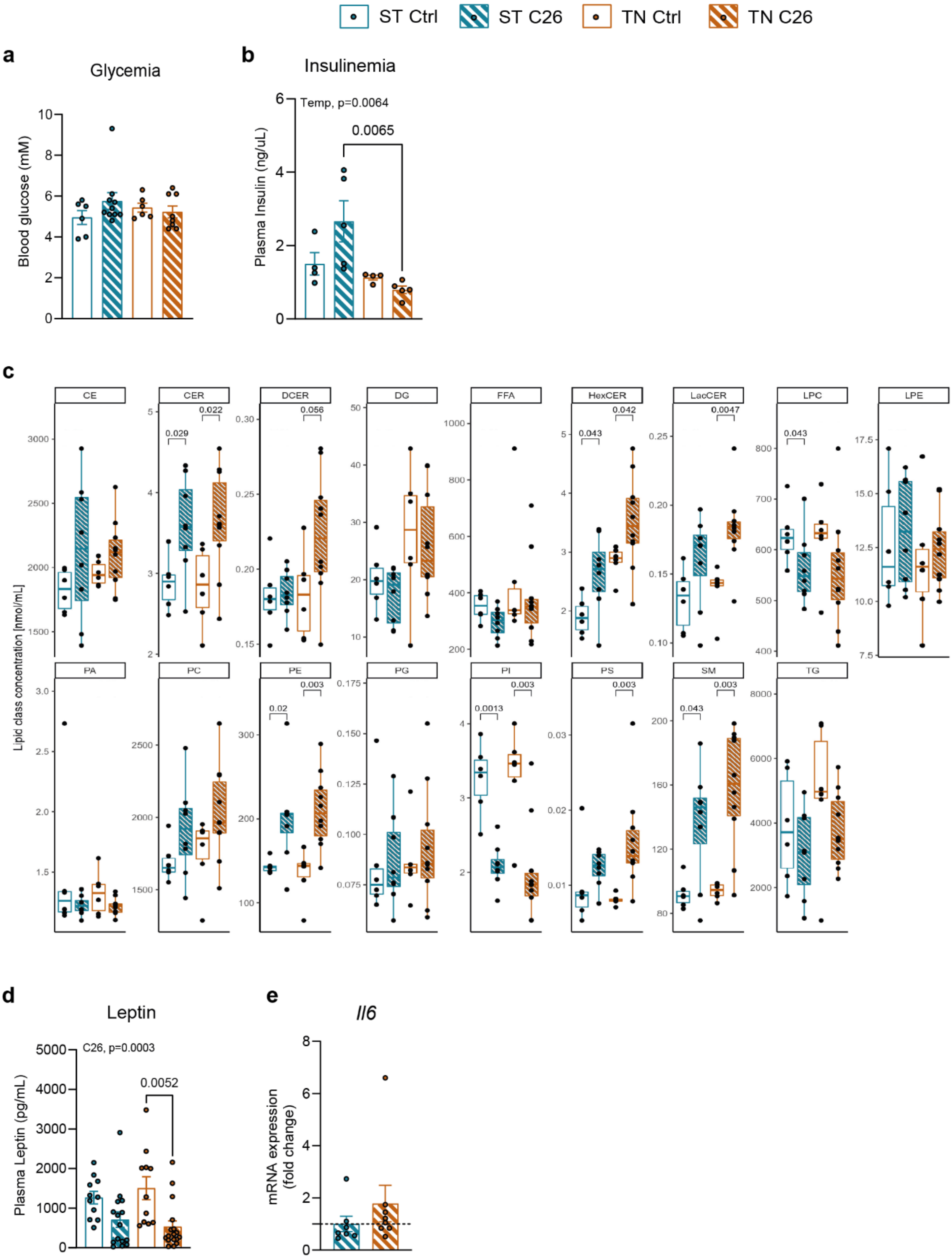

Supplementary Figure 2. (**a**) Fasting blood glucose. (**b**) Fasting blood insulin. (**c**) Plasma lipid class sum concentration. (**d**) Plasma leptin levels. (**e**) IL-6 gene expression in tumors. Data are expressed as mean ± SE including individual values where applicable. (**a, b, d**) Two-way ANOVA test with Tukey's post-hoc test. (**c**) Wilcoxon test. (**e**) Student's T-test. C26, main cancer effect; Temp, main temperature effect.

Supplementary Figure 3

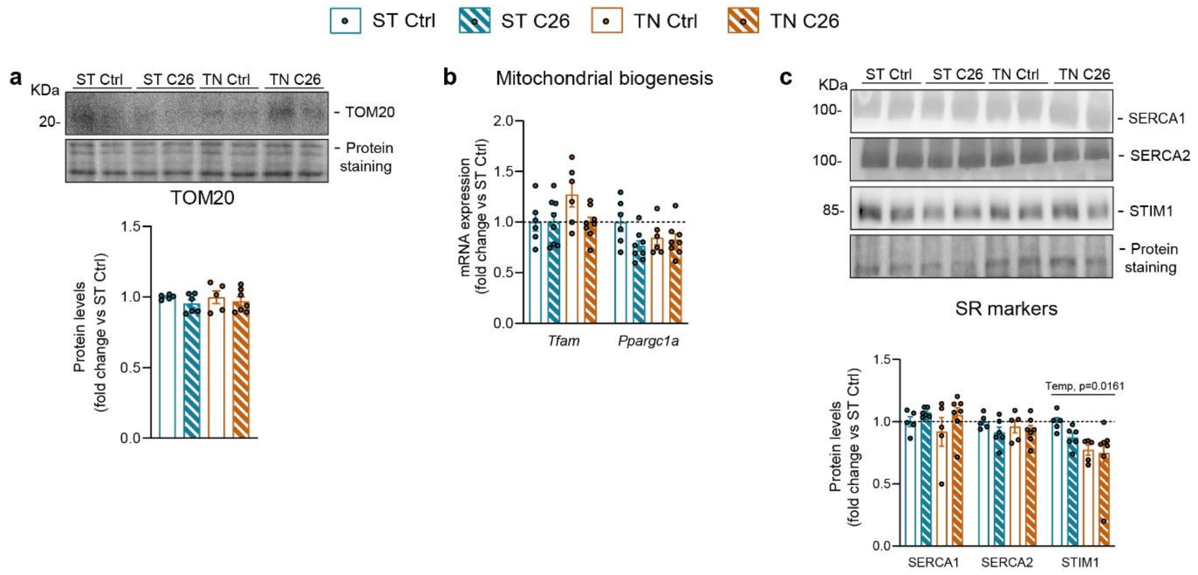

Supplementary Figure 3. (**a**) Representative immunoblot of TOM20 and band quantification in SkM. (**b**) *Tfam* and *Ppargc1a* mRNA levels SkM. (**c**) Representative immunoblots of SR markers and band quantification in SkM. Data are expressed as mean ± SE including individual values where applicable. (**a-c**) Two-way ANOVA test with Tukey's post-hoc test. Temp, main temperature effect.

Supplementary Figure 4

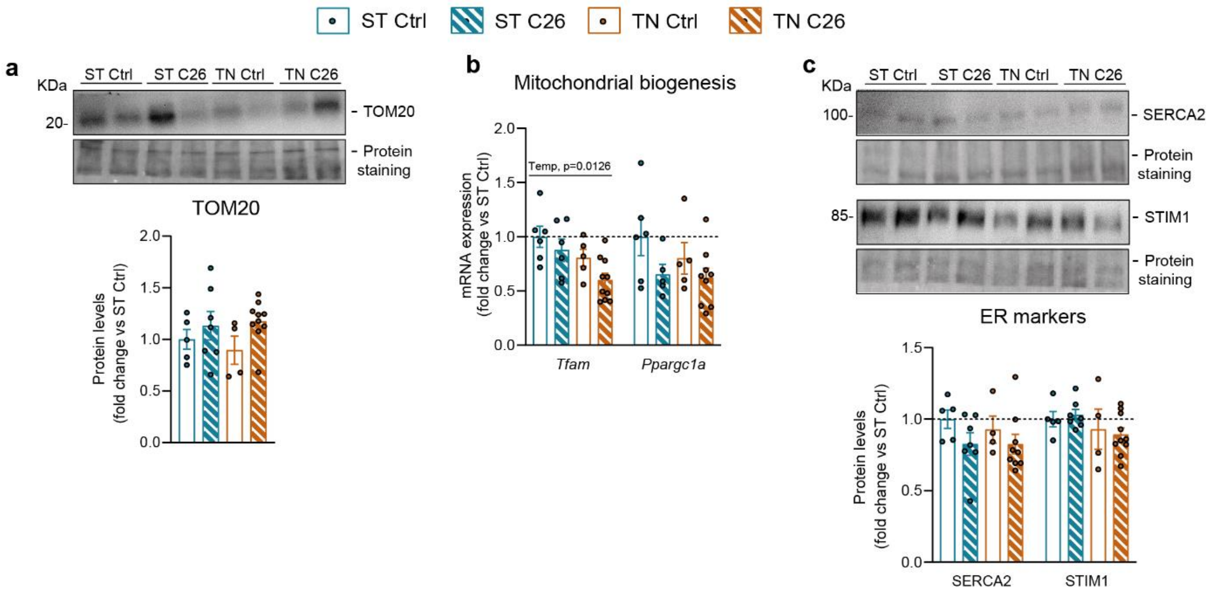

Supplementary Figure 4. (**b**) Representative immunoblot of TOM20 and band quantification in BAT. (**b**) *Tfam* and *Ppargc1a* mRNA levels BAT. (**c**) Representative immunoblots of ER markers and band quantification in BAT. Data are expressed as mean ± SE including individual values where applicable. (**a-c**) Two-way ANOVA test with Tukey's post-hoc test. Temp, main temperature effect.

Supplementary Table 1: SYBR green mouse primers

Targeted gene	*Forward sequence*	*Reverse sequence*	*Source*
4ebp1	CACGCTCTTCAGCACCAC	GGAGGCTCATCGCTGGTAG	Ninfali et al 2018 doi: 10.1093/nar/gky835
Fbxo32	GCAAACACTGCCACATTCTCTC	CTTGAGGGGAAAGTGAGACG	Ninfali et al 2018 doi: 10.1093/nar/gky835
Trim63	TGTCTGGAGGTCGTTTCCG	ATGCCGGTCCATGATCACTT	Ninfali et al 2018 doi: 10.1093/nar/gky835
Slc25a7	AGGCTTCCAGTACCATTAGGT	CTGAGTGAGGCAAAGCTGATTT	Rupar et al 2023 doi: 10.1111/febs.16716
Cebpa	CAAGAACAGCAACGAGTACCG	GTCACTGGTCAACTCCAGCAC	Primerbank
Cepbd	CGACTTCAGCGCCTACATTGA	CTAGCGACAGACCCCACAC	Primerbank
Leptin	GAGACCCCTGTGTCGGTTC	CTGCGTGTGTGAAATGTCATTG	Primerbank
Dio2	AGTCAAGAAGGTGGCATTCG	ACAGCTTCCTCCTAGATGCCT	Yoshizawa et al 2022 doi: 10.1038/s41467-022-35219-z
Elvol3	TTGGGGATAGGGGGTGTGTG	TCTCCCCTCCCCTCCAAGTC	Yoshizawa et al 2022 doi: 10.1038/s41467-022-35219-z
Sirt7	TGCCAGGCACTTGGTTGTCT	TAGGCTCCGCTTCGCTTAGG	Yoshizawa et al 2022 doi: 10.1038/s41467-022-35219-z
Il6	TAGTCCTTCCTACCCCAATTTCC	TTGGTCCTTAGCCACTCCTTC	Irazoki et al 2023 doi: 10.1038/s41467-022-35732-1
Tfam	ATTCCGAAGTGTTTTTCCAGCA	TCTGAAAGTTTTGCATCTGGGT	Primerbank
Ppargc1a	TATGGAGTGACATAGAGTGTGCT	CCACTTCAATCCACCCAGAAAG	Primerbank
Atp2a1	TGTTTGTCCTATTTCGGGGTG	AATCCGCACAAGCAGGTCTTC	Primerbank
Atp2a2	GAGAACGCTCACACAAAGACC	CAATTCGTTGGAGCCCCAT	Primerbank
Sln	GCTCCTCTTCAGGAAGTGAAG	TGGCCCCTCAGTATTGGTAGG	Pant et al 2015 doi: 10.1242/jeb.119164
b-actin	GGTCATCACTATTGGCAACGA	GTCAGCAATGCCTGGGTACA	Irazoki et al 2023 doi: 10.1038/s41467-022-35732-1
36b4	TCATCCAGCAGGTGTTTGACA	GGCACCGAGGCAACAGTT	Self-designed

Supplementary Table 2: Primary antibodies

Target	*Catalog no.*	*Company*	*Dilution*
Anti-OXPHOS cocktail	MS604-300	Abcam	1:1000
Anti-UCP1	Ab155117	Abcam	1:1000
Anti-TOM20	Sc-17764	Santa Cruz Biotechnology	1:1000
Anti-SERCA1	MA3-912	Invitrogen	1:1000
Anti-SERCA2	MA3-910	Invitrogen	1:1000
Anti-STIM1	4916	Cell Signaling	1:1000
